# G-quartet driven adaptive hydrogels that integrate constitutional dynamics for antibacterial action and tissue repair

**DOI:** 10.1016/j.isci.2026.116107

**Published:** 2026-06-09

**Authors:** Sudeshna Mondal, Binayak Lala, Debasmita Biswas, Jean Marie Lehn, Jyotirmayee Dash

**Affiliations:** 1 School of Chemical Sciences, Indian Association for the Cultivation of Science, 2A and 2B Raja S.C. Mullick Road, Jadavpur, Kolkata 700032, India; 2 Laboratoire de Chimie Supramoléculaire, Institut de Science et d’Ingénierie Supramoléculaires (ISIS), Université de Strasbourg, 8 allée Gaspard Monge, 67000 Strasbourg, France

**Keywords:** biomedical engineering, tissue engineering, biomaterials, biomedical materials

## Abstract

We report a nucleic acid inspired constitutional dynamic hydrogel (G4TZ) self-assembled from guanosine, a phenylboronic acid, and a thiazole peptide through dynamic covalent linkages formed by boronate ester and imine bonds, together with supramolecular interactions involving π–π stacking and K^+^-induced G-quartet formation. The resulting transparent and injectable hydrogel exhibits shear-thinning and pH-responsive behavior. G4TZ displays potent antibacterial activity against multidrug-resistant *Streptococcus pneumoniae* through reactive oxygen species generation, membrane disruption, DNA fragmentation, and biofilm inhibition, while maintaining excellent cytocompatibility. Furthermore, it accelerates re-epithelialization and promotes wound healing both *in vitro* and *in vivo*. This dynamic covalent and supramolecular system unites molecular design with biological function, offering strong translational potential for antimicrobial and regenerative medicine. More broadly, this work demonstrates reaction-driven multicomponent self-assembly as a general strategy to engineer multifunctional biomaterials with enhanced stability, biocompatibility, and therapeutic efficacy.

## Highlights

• Programmable nucleoside hydrogel with dynamic covalent and supramolecular chemistry
• Combats MDR pathogens via ROS, membrane rupture, DNA damage and biofilm inhibition
• Shows excellent biocompatibility with injectable, self-healing properties for wound healing
• Dynamic self-assembly bridges molecular design with next-generation therapeutics

## Graphical abstract

**Figure:**
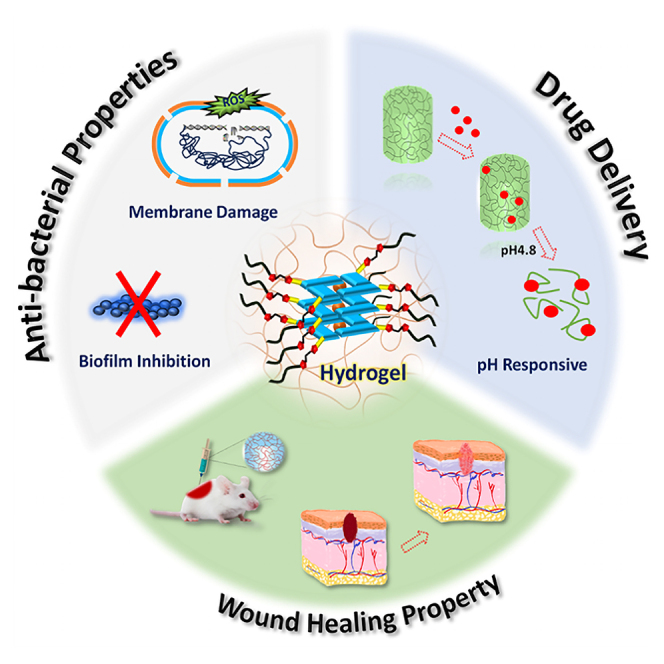

## Introduction

The rapid rise of antimicrobial resistance (AMR) has created an urgent need for novel therapeutic strategies that function beyond conventional enzyme and protein targeted antibiotics, which are increasingly undermined by mutations, efflux pumps, and biofilm-mediated protection.^1^^,^^2^^,^^3^ To address this challenge, supramolecular hydrogels have emerged as promising soft materials with tunable properties such as injectability, porosity, and responsiveness to pH or ionic strength.^4^^,^^5^^,^^6^^,^^7^^,^^8^^,^^9^ Unlike traditional drugs, these materials can disrupt microbial membranes directly, reducing the risk of resistance.^10^

Dynamic covalent chemistry (DCC) offers unique opportunities for constructing adaptive materials through reversible covalent bonds such as imines and boronate esters.^11^^,^^12^ In hydrogels, these interactions endow networks with structural adaptability, self-healing, and stability under physiological conditions, while also supporting biological processes such as wound repair.^13^ Despite these advances, most current antimicrobial hydrogels rely on metallic nanoparticles or drug cargos, which are often hampered by cytotoxicity, poor stability, and limited translational potential.^14^

To overcome these limitations, attention has shifted toward intrinsically antimicrobial supramolecular hydrogels composed of biocompatible low-molecular-weight gelators (LMWGs).^15^^,^^16^ Peptide-based LMWGs offer biodegradability and structural versatility,^17^ while nucleobases such as guanosine form highly ordered G-quartets and G-quadruplexes through Hoogsteen hydrogen bonding and π–π stacking, stabilized by monovalent cations.^18^^,^^19^ These unique assemblies have inspired functional soft materials ranging from ion channels to liquid crystals and gels.^20^^,^^21^^,^^22^^,^^23^ However, synthetic G-quadruplex gels often suffer from instability, dilution-induced disassembly and crystallization.^24^^,^^25^^,^^26^ To enhance the stability and functionality of these systems, strategies such as boronic ester formation, co-assembly with aromatic linkers, and molecular substitution at key positions of the guanine ring have been explored.^27^^,^^28^^,^^29^^,^^30^

Building on these concepts, we herein report a dynamic covalent supramolecular hydrogel (G4TZ) formed by conjugating guanosine with a thiazole-containing peptide using an aldehyde functionalized phenylboronic acid (2-FPBA) as a linker. This system integrates the structural sophistication of G-quadruplex assemblies with the antimicrobial potency of thiazole peptides, producing an injectable, stimuli-responsive, and mechanically robust hydrogel. Mechanistic studies reveal potent antibacterial activity against multidrug-resistant *Streptococcus pneumoniae* through reactive oxygen species (ROS) generation, DNA damage, membrane disruption, and biofilm inhibition, alongside accelerated wound healing. Our work establishes a paradigm in programmable, DNA-inspired dynamic covalent hydrogels, uniting nucleobase self-assembly with peptide chemistry to deliver multifunctional antimicrobial and regenerative properties.

## Results

### Dynamic chemistry enables gelation

**Scheme 1:**
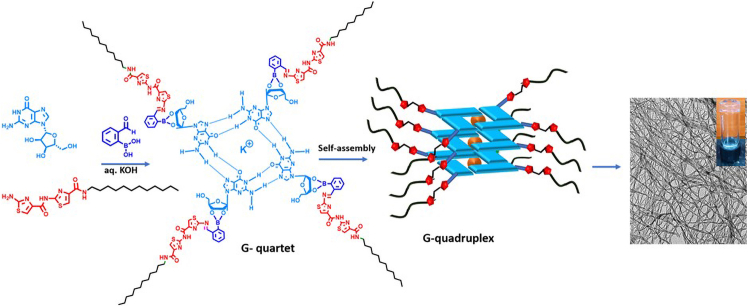
Schematic representation of the formation of a bis-thiazole conjugated G-quadruplex hydrogel

We designed a constitutional dynamic covalent system comprising guanosine, a phenylboronic acid derivative, and a thiazole-based peptide to construct a self-assembled hydrogel framework (Scheme 1 and Scheme S1).Hydrogel formation is driven by two orthogonal dynamic covalent reactions: (1) reversible boronate ester linkages between the *cis*-diol groups of guanosines and the boronic acid,^31^ and (2) pH-sensitive imine (Schiff base) bonds formed between the aldehyde group of boronic acid and the amine of the peptide. Together, these equilibrating reactions result in nucleoside-heteroaromatic compound conjugates. Upon heating to 85°C in the presence of KOH and subsequent cooling, spontaneous self-assembly occurs, producing a transparent and stable hydrogel.

Beyond covalent interactions, the hydrogel architecture is stabilized by multiple noncovalent forces. Potassium ion-templated G-quartets, stabilized by Hoogsteen hydrogen bonding among guanosine bases, stack through π–π interactions to form extended fibrous G-quadruplex networks. Incorporation of a bis-thiazole amide bearing a hydrophobic chain further reinforces this framework: the aromatic thiazole rings contribute additional π–π stacking, while the amide groups engage in hydrogen bonding that promotes inter-fiber association. The hydrophobic dodecylamine tail enhances lateral bundling and fiber entanglement, thereby improving viscoelasticity and reducing solubility.^32^

Control experiments confirm the importance of the thiazole amides in the hydrogel network; boronic acids alone can form G-quartet-based gels via boronate ester crosslinking, but the resulting hydrogel, particularly formed with aldehyde functionalized boronic acid is weak and unstable (Figure S3).^23^ In contrast, incorporation of bis-thiazole amide provides complementary supramolecular interactions, leading to a mechanically robust, injectable G4 hydrogel with enhanced stability and properties suitable for biomedical applications.

### Viscoelastic and self-healing properties

**Figure 1:**
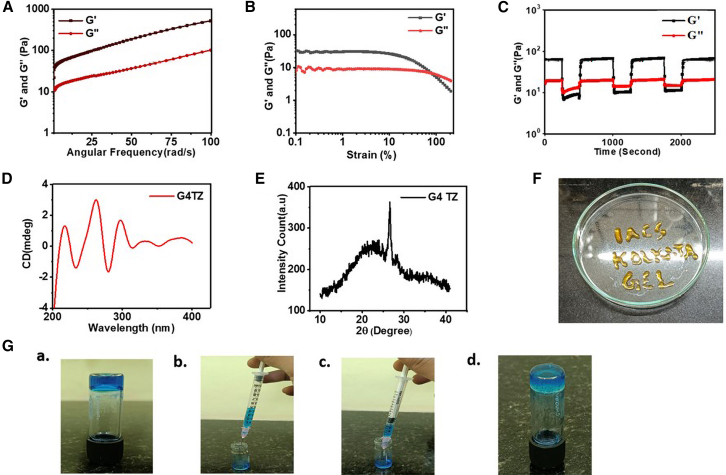
Rheological and structural characterization of the G4TZ hydrogel (A) Frequency sweep. (B) amplitude sweep. (C) time-dependent step-strain sweep demonstrating viscoelastic and thixotropic properties. (D) CD spectrum confirming G-quadruplex formation. (E) PXRD pattern indicating π–π stacking of G-quartets in the hydrogel fibers. (F) Printability of methyl orange-doped G4TZ gel through a 24 G needle. (G) Sequential images demonstrating the reversible sol-gel transition of the G4TZ hydrogel upon mechanical stress. (a) Methylene blue-doped G4TZ hydrogel in the gel state. (b and c) Disruption of the gel network by loading into a syringe and subsequent injection into a vial. (d) Instantaneous recovery of the self-supporting hydrogel, confirming its thixotropic and rapid self-healing behavior.

To investigate the viscoelastic properties, stiffness, and mechano-responsiveness of the G4TZ hydrogel, rheological experiments were performed.^33^ In the frequency sweep, the storage modulus (G′) consistently exceeded the loss modulus (G″) across the measured range, confirming gel-like viscoelasticity (Figure 1A). The gradual decrease in G′ with decreasing angular frequency (ω) suggested that gelation is governed by physical crosslinking. In the amplitude sweep at 10 rad/s, G′ and G″ remained constant up to 25% strain, reflecting an intact network, before intersecting at ∼70% strain, indicative of a gel-to-sol transition (Figure 1B). In contrast, the guanosine/boronic acid gel alone showed an intersection at ∼18% strain, highlighting the superior mechanical robustness of G4TZ (Figure S8).Dynamic step-strain experiments further revealed thixotropic behavior and self-healing properties. At low strain (1%), G′ > G″ confirmed a gel state, while at high strain (100%), G″ > G′ indicated sol disruption. Upon returning to low strain, the gel rapidly recovered, demonstrating reversible sol-gel transitions and excellent network recovery (Figure 1C).

This shear-thinning property rendered G4TZ injectable and printable.^34^ As shown in Figure 1F, methyl orange-doped G4TZ was extruded smoothly through a 24 G needle to print the text “IACS KOLKATA GEL”, demonstrating its printability for 3D bioprinting and drug delivery applications. Similarly, methylene blue-doped hydrogel readily transitioned to a sol under applied pressure during injection and reverted to a gel on exit, with rapid shape recovery confirmed by vial inversion (Figure 1G).

Structural analysis validated the supramolecular architecture. Circular dichroism (CD) spectroscopy revealed positive signals at ∼297 and ∼263 nm, and negative peaks at ∼278 and ∼235 nm, characteristic of G-quadruplex topologies in mixed head-to-head and head-to-tail stacking modes (Figure 1D).^35^ Powder X-ray diffraction (PXRD) further confirmed ordered stacking, showing a peak at 26.62° (d = 0.33 nm) corresponding to π–π interactions between G-quartets (Figure 1E).^27^ This feature was absent in the individual components (Figure S4), demonstrating that ordered G-quadruplex formation occurs only upon hydrogel self-assembly.

Rheological and structural analyses establish G4TZ as a mechanically stable, shear-thinning, injectable, and self-healing hydrogel, whose supramolecular architecture is stabilized by both dynamic covalent and non-covalent interactions.

### G-quadruplex and dynamic covalent interplay

**Figure 2:**
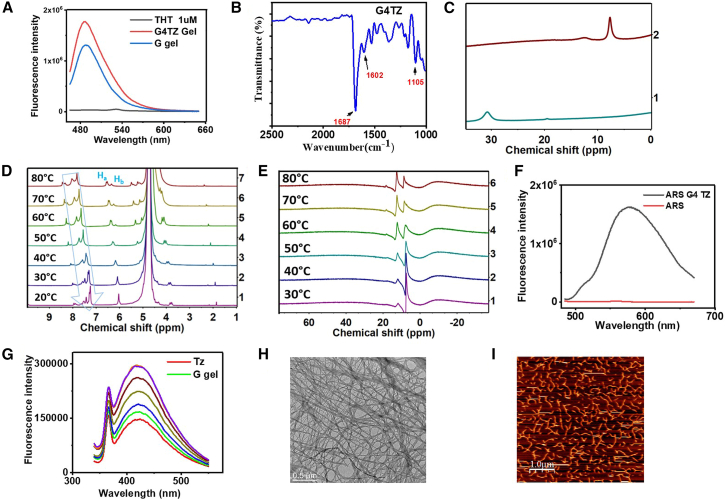
Spectroscopic and microscopic characterization of the G4TZ hydrogel (A) Fluorescence emission spectra of 1 μM ThT in the presence of G-gel and G4TZ gel. (B) FT-IR spectra of G4TZ gel. (C) 11B NMR spectra of G4TZ gel and 2-FPBA. (D) Variable-temperature ^1^H NMR spectra of the G4TZ hydrogel recorded over a temperature range of 20°C–80°C. (E) Variable-temperature ^11^B NMR spectra of the G4TZ hydrogel recorded over a temperature range of 30°C–80°C. (F) Fluorescence emission spectra of ARS in solution and ARS embedded G4TZ gel. (G) Fluorescence response of thiazole peptide TZ with guanosine (G) gel in 100 mM Tris-KCl buffer. (H) TEM image of G4TZ gel showing the formation of an entangled nanofibrillar network. Scale bars, 0.5 μm. (I) AFM image of G4TZ gel. Scale bars, 1 μm.

The formation of G-quadruplex structures in G4TZ was examined using Thioflavin T (ThT), a fluorophore that selectively binds to G-quadruplexes.^36^ Upon addition of the hydrogel, ThT fluorescence at 485 nm increased markedly, confirming the presence of G-quadruplexes within the gel matrix (Figure 2A).

To probe the gelation process, Fourier transform infrared (FT-IR) and ^11^B NMR spectroscopy were employed (Figures 2B and 2C). In the FT-IR spectrum of guanosine, the characteristic C–O–H stretching band at 1082 cm^−1^ disappeared in the hydrogel, consistent with participation in hydrogen bonding or coordination (Figure S5). Likewise, the guanosine C=O stretching peak shifted from 1730 cm^−1^ to 1687 cm^−1^, indicating hydrogen-bonded G-quartet formation.

For the boronic acid, the free aldehyde and B–OH bands at 1668 and 1351 cm^−1^, respectively, were altered upon gelation. A new band at 1105 cm^−1^ signaled B–O–C bond formation, while the aldehyde band shifted from 1668 cm^−1^ to 1602 cm^−1^, consistent with imine (Schiff base) formation between boronic acid and the thiazole derivative. The reduced intensity of the –NH_2_ stretching at 3340 cm^−1^ further corroborated imine formation. These spectral changes confirm the *in situ* generation of imino-boronate linkages as the dynamic covalent crosslinks driving gel assembly. Thus, orthogonal dynamic covalent interactions (boronate ester and imine formation), combined with supramolecular G-quadruplex stacking, establish the multicomponent framework that stabilizes the G4TZ hydrogel.

In parallel, the ^11^B NMR spectrum of free boronic acid in D_2_O showed a sharp resonance at δ ∼30 ppm, characteristic of the free B–OH group. In contrast, the spectrum of G4TZ prepared in 100 mM KOH exhibited an upfield shift to δ ∼8 ppm, reflecting a markedly different boron environment due to boronate ester bond formation, arising from nucleophilic hydroxide addition to the carbonyl group.

To further probe the gelation process, variable-temperature (VT) ^1^H NMR spectra were recorded. As the temperature decreased from 80°C to 20°C, aromatic proton signals (7–8 ppm) shifted upfield, consistent with enhanced shielding from π–π stacking interactions. This indicates that cooling promotes supramolecular aggregation involving guanosine and phenylboronic units. In parallel, integrals corresponding to guanosine (Hb), phenylboronate ester (Ha), and aromatic protons progressively decreased upon cooling, supporting temperature driven molecular association and boronate ester formation (Figure 2D).

VT ^11^B NMR (30°C–80°C) further confirmed dynamic covalent interactions (Figure 2E). As temperature increased, the signal assigned to intramolecular boronate esters (∼7–8 ppm) diminished, while a new resonance at 12–13 ppm emerged, indicating gradual formation of intermolecular boronate esters.^37^ This thermal transformation highlights the role of dynamic boronate ester linkages in maintaining the structural integrity and adaptability of the G4TZ hydrogel.

Finally, fluorescence spectroscopy with alizarin red S (ARS) provided additional support for boronate-based interactions. While ARS alone was nonfluorescent, ARS embedded in G4TZ exhibited a strong emission band at 577 nm, confirming strong binding between ARS and 2-FPBA and corroborating the presence of boronate ester linkages within the hydrogel (Figure 2F).

### Molecular recognition and responsiveness

Thiazole derivatives are known to strongly interact with G-quadruplex structures.^38^ In our earlier work, we reported antiproliferative thiazole-based peptidomimetics that bound terminal G-quartets and capping structures at the 5′/3′ ends of G-quadruplexes, effectively stabilizing these DNA secondary structures.^39^^,^^40^ Guided by this, we hypothesized that the crescent-shaped thiazole peptide (TZ) would selectively interact with G-quadruplex assemblies in G4TZ. Fluorometric titrations confirmed this interaction; TZ displayed a turn-on fluorescence at ∼420 nm upon binding to the G-gel, with intensity increasing sharply upon addition of 1–5 equivalents of gel (relative to guanosine content), indicating strong and specific association with the G-quartet fibers (Figures 2G and S7).

The hydrogel morphology was visualized by TEM and atomic force microscopy (AFM), which revealed a densely entangled nanofibrous network spanning the gel matrix (Figures 2H, 2I, and S6), characteristic of supramolecular gels. This fibrous architecture likely contributes to the mechanical stability and responsiveness of the hydrogel.

Finally, the environment-sensitive drug delivery ability of G4TZ was evaluated by encapsulating doxorubicin (100 μM) and monitoring its release at different pH values. Negligible release was observed at physiological pH 7.4, whereas acidic pH (4.8) triggered substantial release, and alkaline pH (8.8) resulted in slow, sustained release (Figure S9). These findings demonstrate the pH-responsive drug release behavior of G4TZ, attributed to destabilization or partial degradation of the nanofibrous network in non-physiological environments.^41^

### Cellular uptake and antibacterial effects

Inherently antibacterial hydrogels have gained attention as advanced biomaterials for preventing biofilm formation and treating wound infections.^42^ Our G4TZ hydrogel incorporates a thiazole-based peptide, featuring a thiazole dimer at the N-terminus and a dodecylamine group at the C-terminus, lacking conventional cationic residues such as arginine or lysine. While antimicrobial peptides (AMPs) are promising alternatives to traditional antibiotics, their clinical use is limited by poor selectivity, cytotoxicity, short half-life, instability, and high production costs.^43^ AMP loaded hydrogels have been investigated as delivery platforms to overcome these limitations; however, they often demand high AMP loadings, which can increase cytotoxicity and production costs.

To assess the antibacterial efficacy of G4TZ, we performed minimum inhibitory concentration (MIC) assays against clinically isolated Gram-negative (*Escherichia coli, Acinetobacter*) and Gram-positive (*Staphylococcus aureus, Streptococcus pneumoniae*) bacteria with strain identification carried out using the VITEK MALDI-TOF MS system (Biomerieux). Remarkably, G4TZ exhibited potent antibacterial activity against multidrug-resistant *S. pneumoniae* with a low MIC of 20 μg/mL (Table 1). In contrast, neither the guanosine-boronic acid gel (G-gel) nor the thiazole peptide (TZ) alone displayed measurable activity against these resistant strains.

**Table 1:** The MIC values of G gel, TZ and G4TZ gel against a range of pathogenic clinical isolates of *E. coli*, *A. baumanii*, *S. aureus*, and *S. pneumoniae.*

	*E. coli* (μg/mL)	*A. baumanii* (μg/mL)	*S. aureus* (μg/mL)	*S. pneumoniae* (μg/mL)
G gel	>100	>100	>100	>100
TZ	>100	>100	>100	>100
G4TZ	>100	>100	>100	20

These results show that antibacterial activity arises only when the bis-thiazole motif is integrated into the G-quadruplex hydrogel framework, indicating that G4TZ acts as an intrinsically antimicrobial material rather than as a passive carrier. While the individual components are inactive, their assembly into the G4 hydrogel creates an adaptive interface that enables effective interaction with bacterial membranes. The dynamic covalent groups at the hydrogel surface allow multivalent and reversible binding to membrane components of *S. pneumoniae*. These interactions could facilitate localized binding, membrane insertion of the thiazole motif, and subsequent membrane disruption, leading to bacterial death. The pH-responsive nature of the imine and boronate ester linkages further enhances antibacterial activity under the locally acidic conditions generated by *S. pneumoniae*.

**Figure 3:**
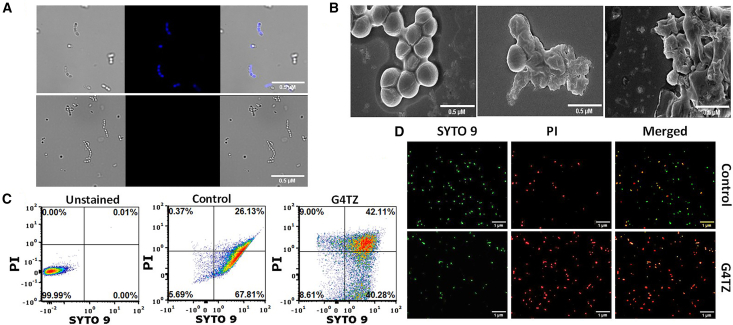
G4TZ induced membrane damage in *S. pneumoniae* (A) Confocal images of *S. pneumoniae* showing cellular permeability of TZ, top; (1) bright field image of *S. pneimoniae*, (2) image of *S. pneumoniae* in blue filter, and (3) merged image of representations 1 and 2. Bottom image G4TZ; (1) bright field image of *S. pneumoniae*, (2) image of *S. pneumoniae* in blue filter and (3) merged image of representations 1 and 2. Scale bars, 0.5 μm. (B) SEM images showing altered morphology of *S. pneimoniae* after treatment with G4TZ, left untreated representation, middle representation G4TZ 2 μg/mL and right representation G4TZ 4 μg/mL. Scale bars, 0.5 μm. (C) Quantitative PI/SYTO-9 flow cytometry reveals a marked shift from SYTO-9^+^/PI^−^ membrane-intact cells in control samples to predominantly PI-positive, membrane damaged populations upon G4TZ treatment, confirming bacterial membrane permeabilization. (D) Representative live/dead fluorescence microscopy images of control and G4TZ treated *S. pneumoniae*. Healthy bacteria with intact membranes stain green (SYTO-9^+^), while bacteria with damaged membranes that permit entry of propidium iodide stain red (PI^+^). G4TZ treatment causes a clear shift from a predominantly green (live) population to a red (dead/damaged) population. Scale bars, 1 μm.

Confocal laser scanning microscopy supports this mechanism by confirming the selective association of G4TZ with *S. pneumoniae*. A strong fluorescence signal is observed on bacteria treated with the hydrogel, whereas no signal is detected in *S. pneumoniae* treated with the TZ peptide alone (Figure 3A).

### Antibacterial mechanism

Having established the selective association of G4TZ with *S. pneumoniae* and its potent antibacterial efficacy, we next elucidated the mechanistic basis underlying this activity. Scanning electron microscopy (SEM) analysis revealed that untreated bacteria exhibited smooth, intact morphologies, whereas G4TZ treated cells displayed pronounced surface deformation, membrane rupture, and extensive fragmentation, indicating localized disruption of the cell wall and membrane (Figure 3B).

Consistent with the SEM observations, live/dead fluorescence imaging revealed a pronounced shift from SYTO-9 positive (green) viable cells in the control group to propidium iodide (PI) positive (red) cells following G4TZ treatment, indicating severe loss of membrane integrity (Figure 3D). Quantitative analysis by flow cytometry using SYTO-9/PI staining further confirmed this effect, showing that ∼70% of control bacteria retained intact membranes, whereas G4TZ treatment severely damaged the membrane in ∼50% of the bacterial population (Figure 3C).

**Figure 4:**
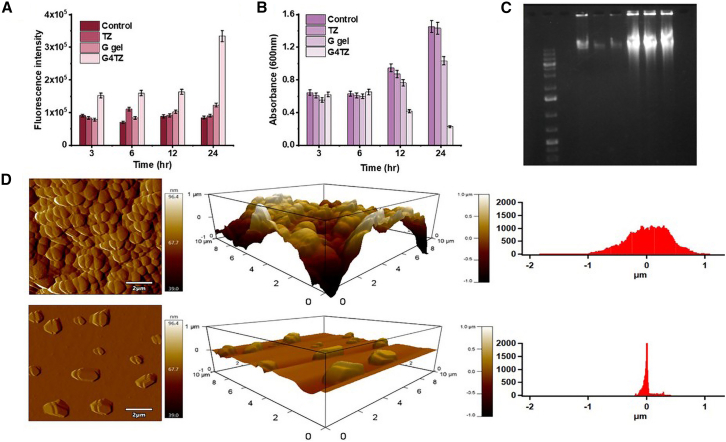
Multimodal antibacterial mechanism of G4TZ against *S. pneumoniae* (A) Intracellular ROS levels in *S. pneumoniae* after treatment with G4TZ. Error bars represent ±5% of the mean (mean ± SD, *n* = 3). (B) Time-dependent bacterial growth (OD_600_) after treatment with TZ, G gel, and G4TZ, showing significant growth inhibition in the presence of G4TZ compared to controls. Error bars represent ±5% of the mean (mean ± SD, *n* = 3). (C) Agarose gel electrophoresis showing genomic DNA fragmentation: lane 1; 1 kb DNA ladder, lane 2; control, lane 3; TZ 2 μM, lane 4; TZ 4 μM, lane 5; G4TZ 1 μg/mL, lane 6; G4TZ 2 μg/mL, lane 7; G4TZ 3 μg/mL. (D) AFM height images, three-dimensional topography, and histogram profiles of *S. pneumoniae* biofilms: top image, untreated control; bottom image, after G4TZ treatment. Scale bars, 2 μm.

To establish a quantitative temporal relationship in antibacterial activity, we performed parallel time-resolved measurements of bacterial viability and intracellular ROS levels over 24 h. Control and TZ treated cells exhibited consistently low ROS levels at all time points. In contrast, G4TZ treated bacteria showed a modest increase in ROS as early as 3 and 6 h post-exposure (Figure 4A), a time point at which bacterial viability remained largely unaffected (Figure 4B). ROS levels increased progressively at 12 h and peaked at 24 h, corresponding to near-complete growth inhibition. This temporal progression indicates that ROS accumulation precedes and contributes to bacterial cell death, rather than arising as an immediate consequence of membrane disruption.

ROS are well known to damage essential biomolecules, including DNA and nucleotide pools. To probe whether G4TZ-induced ROS contributes to DNA damage, genomic DNA integrity was analyzed after 24 h hydrogel treatment. Agarose gel electrophoresis revealed marked DNA fragmentation in *S.pneumoniae* treated with 1 μg/mL (C1), 2 μg/mL (C2), and 3 μg/mL (C3) of G4TZ, whereas untreated controls and TZ treated bacteria retained intact genomic DNA (Figure 4C).

These results indicate that sustained oxidative stress induced by G4TZ leads to lethal DNA damage. To translate these mechanistic insights into a clinically relevant context, we evaluated the efficacy of G4TZ against *S. pneumoniae* biofilms, which pose a major therapeutic challenge due to their structural integrity and inherent resistance to antibacterial agents.

AFM revealed that untreated *S. pneumoniae* biofilms exhibited smooth, compact surfaces with intact extracellular polymeric substance (EPS) layers and uniform organization, indicating preserved structural integrity. In contrast, G4TZ treated biofilms displayed structural disruption, including collapsed cells, membrane blebbing, cellular debris, EPS fragmentation, and leakage of intracellular contents (Figure 4D). Quantitative AFM analysis further revealed a substantial reduction in biofilm thickness (from 613.43 to 322.30 nm) and surface roughness (from 471.70 to 170.01 nm), confirming effective destabilization of the biofilm matrix.

Finally, cytocompatibility was assessed in human hepatic (WRL 68) and keratinocyte (HaCaT) cell lines. G4TZ showed no detectable cytotoxicity at concentrations up to 100 μg mL^−1^ (Figure S10), confirming its suitability for potential biomedical applications.

Thus, G4TZ operates through a multi-step antibacterial mechanism involving (1) selective association with the target bacterium, (2) membrane disruption, (3) sustained ROS generation that temporally correlates with loss of bacterial viability, and (4) ROS-mediated DNA damage accompanied by biofilm collapse. This combination of pathogen selectivity, a quantitatively supported mechanism, potent antibacterial activity, and host compatibility supports the potential of G4TZ as a programmable antimicrobial biomaterial with translational relevance.

### Promotion of wound healing

**Figure 5:**
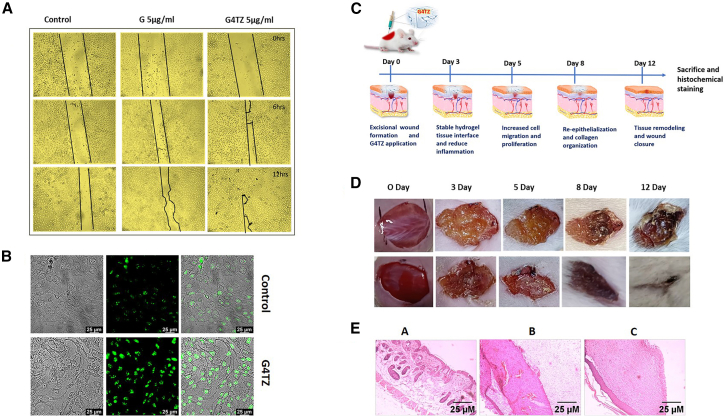
*In vitro* and *in vivo* evaluation of G4TZ hydrogel in wound healing (A) Scratch wound healing assay of HaCaT cells treated with guanosine (G) gel and G4TZ gel. Representative images were captured at 0-, 6-, and 12-h post-wound creation to assess cell migration and wound closure. (B) Ki67 immunofluorescence staining reveals keratinocyte proliferation in HaCaT cells. Representative images of untreated (control, top panel) and G4TZ treated (bottom panel) HaCaT cells, where Ki67-positive nuclei (green) indicate proliferating cells. Scale bars, 25 μm. (C) Schematic illustration of G4TZ-mediated wound healing. (D) *In vivo* wound healing evaluation. Top representation, images of wounds of control groups on days 0, 3, 5, 8 and 12. Bottom panel, images of wounds of G4TZ treated groups on days 0, 3, 5, 8, and 12. (E) Histological comparison of skin wound healing of untreated and G4TZ treated mice skin. Representative H&E-stained skin sections showing (A) healthy skin architecture with intact epidermis, dermis, and visible skin appendages such as hair follicles and sebaceous glands. (B) Untreated wound area displays disorganized tissue architecture, absence of epidermal layer, and dense inflammatory cell infiltration. (C) G4TZ treated wounded skin at a later stage of healing, displaying mature granulation tissue, reduced cellularity, and enhanced collagen deposition indicative of early scar formation. Scale bars, 25 μm.

Building on the intrinsic antibacterial activity of the G4TZ network, we next examined whether its adaptive viscoelastic properties contribute to enhanced wound healing. Bacterial infection and mechanical instability are major factors that delay tissue repair; therefore, hydrogels that combine antibacterial function with self-healing and viscoelastic behavior are well suited for wound healing applications.^44^^,^^45^ Rheological and injectability studies show that molecular reversibility in G4TZ gives rise to thixotropic, self-healing viscoelasticity, which is important in the mechanically dynamic wound environment. We therefore evaluated the regenerative ability of G4TZ in keratinocyte cultures and *in vivo* wound models. In HaCaT scratch assays, G4TZ treatment markedly enhanced keratinocyte migration and promoted faster gap closure compared with untreated controls (Figure 5A). To further understand the biological basis of this enhanced wound closure, keratinocyte proliferation was assessed using Ki67 immunofluorescence staining. G4TZ treated cells showed a pronounced increase in Ki67 positive nuclei and proliferative foci compared with untreated controls (Figure 5B), indicating that wound closure results from the combined effects of increased cell migration and proliferation.

In a BALB/c excisional wound model, G4TZ-treated wounds healed more rapidly, with clear improvement by day 5 and near-complete closure by day 12 (Figures 5C and 5D), whereas the guanosine gel (G gel) did not promote rapid wound healing (Figure S11). Histological analysis supported these findings, demonstrating intact epidermal layers, organized collagen deposition, and reduced inflammation in G4TZ treated wounds, whereas untreated wounds displayed disrupted epidermis, persistent inflammation, and disordered collagen (Figure 5E). These observations suggest that the self-healing nature of G4TZ allows it to remain closely associated with the wound surface during repeated deformation, maintaining its antimicrobial activity and protective function. The adaptive viscoelastic properties of the hydrogel also support cell material interactions that promote keratinocyte migration, proliferation, and matrix remodeling, leading to effective re-epithelialization and tissue regeneration.

## Discussion

The development of multifunctional biomaterials that both combat bacterial infections and promote tissue regeneration is vital for next-generation wound care. Here, we report a self-assembled hydrogel (G4TZ) that integrates dynamic covalent interactions (boronate esters and imines) with supramolecular G-quadruplex assemblies to achieve stability, injectability, and responsiveness. Constructed from guanosine, 2-formylphenylboronic acid, and a crescent-shaped thiazole peptide, G4TZ establishes a robust dynamic network reinforced by π–π stacking and K^+^-templated G-quartet formation.

Functionally, G4TZ displays potent antibacterial activity against multidrug-resistant *Streptococcus pneumoniae* through membrane disruption, ROS generation, DNA fragmentation, and biofilm inhibition, as demonstrated by AFM and SEM. Importantly, the hydrogel is biocompatible and pH-responsive, enabling programmable drug release. *In vitro* and *in vivo* studies further confirmed its ability to accelerate re-epithelialization, promote tissue regeneration, and advance scar maturation, establishing clear translational relevance.

Collectively, this work introduces G4TZ as a double-dynamic (covalent and supramolecular) platform^46^ that bridges molecular design with functional biology. By uniting antimicrobial efficacy and regenerative potential in a single system, G4TZ sets the stage for next-generation constitutional dynamic biomaterials with customizable biological functions and clinical promise.

### Limitations of the current study

The *in vivo* wound healing was mainly evaluated by visual wound assessment and H&E staining. Quantitative analysis of healing kinetics and statistical comparison of closure rates would strengthen the conclusions. Systemic safety was not assessed by histology of major organs. In addition, detailed *in vivo* mechanistic validation and the degradation or stability profile of G4TZ hydrogel under physiological conditions were not fully examined and will be addressed in future studies.

## Resource availability

### Lead contact

Requests for further information and resources should be directed to and will be fulfilled by the lead contact, Jyotirmayee Dash (ocjd@iacs.res.in).

### Materials availability

All unique reagents generated in this study are available from the lead contact upon request.

### Data and code availability

• All data and code underpinning spectra presented herein are available from the lead contact upon request.
• Data: All data supporting the findings of this study are available from the lead contact upon request. Spectroscopic and characterization data (CD, FTIR, NMR, PXRD, and fluorescence, rheology) are included in the published article and supplemental information. Microscopy images (TEM, AFM, SEM, and confocal) and flow cytometry data are available from the lead contact upon request. *In vivo* wound healing data are presented in the main figures. No high-throughput sequencing or repository-based datasets were generated in this study; therefore, no accession codes are applicable.
• Code: No original code was generated during this study. Data analysis was performed using commercial software (OriginPro 8.0, ImageJ, WSxM 5.0) as listed in the key resources table and custom analysis routines described in the method details section.
• Other items: All unique reagents and materials generated in this study, including the G4TZ hydrogel system and thiazole peptide, are available from the lead contact upon reasonable request, subject to standard material transfer agreements.

## Acknowledgments

S. M. thanks UGC for senior research fellowship. B.L. and D.B. thanks CSIR for senior research fellowship. J.D. thanks the DST SERB-CRG project [CRG/2021/004525] and ICMR project [IIRPIG-2024-01-01128] for funding. We thank Aftab Hossain Khan, IACS, Kolkata for his support in performing the rheological experiments.

## Author contributions

J.D. conceived the idea. B.L. carried out the synthesis, FTIR, TEM, rheology, CD spectroscopy, and NMR studies. S.M. performed the fluorescence spectroscopy, AFM, SEM, and other biological experiments. S.M. and D.B. carried out *in vivo* studies. S.M. developed the first draft and J.D. wrote the manuscript. J.M.L. contributed scientific insights during manuscript preparation and critically reviewed the manuscript.

## Declaration of interests

The authors declare no competing interests.

## STAR★Methods

### Key resources table

**Table:** 

REAGENT or RESOURCE	SOURCE	IDENTIFIER
**Antibodies**		

Ki-67 Monoclonal Antibody (SolA15), FITC	Invitrogen	Cat# 11-5698-82; RRID: AB_11151330

**Bacterial strains**		

*Escherichia coli*	Isolated from patient, CNCI, Kolkata	N/A
*Acinetobacter baumannii*	Isolated from patient, CNCI, Kolkata	N/A
*Staphylococcus aureus*	Isolated from patient, CNCI, Kolkata	N/A
*Streptococcus pneumoniae*	Isolated from patient, CNCI, Kolkata	N/A

**Chemicals**		

Guanosine	Sigma-Aldrich	Cat# 75903
Phenylboronic acid (2-FPBA)	Sigma-Aldrich	Cat# 431958
Thioflavin T (ThT)	Sigma-Aldrich	Cat# 596200
DCFDA	Invitrogen	Cat# D399
SYTO 9	Invitrogen	Cat# S34854
Propidium Iodide (PI)	Invitrogen	Cat# P1304MP
Doxorubicin	Sigma-Aldrich	Cat# D5220
XTT [2,3-bis-(2-methoxy-4-nitro-5-sulfophenyl)-2H-tetrazolium-5-carboxanilide]	Sigma-Aldrich	Cat# X4626
Phenazine methosulfate (PMS)	Sigma-Aldrich	Cat# P9625

**Experimental models: Cell lines**		

WRL68 (human hepatic cells)	NCCS	N/A
HaCaT (human keratinocyte)	ATCC	Cat# CRL-2404; RRID: CVCL_0038

**Experimental models: Organisms/strains**		

Mouse: BALB/c	Center for Laboratory Animal Research and Training (CLART), Kalyani	N/A

**Software and algorithms**		

ImageJ	National Institutes of Health	https://imagej.nih.gov/ij/
WSxM 4.0 software	Nanotec Electronica	http://www.wsxm.eu
OriginPro 8.0	OriginLab Corporation	https://www.originlab.com

### Experimental model and study details

#### Bacterial strains

Clinical isolates of *Escherichia coli*, *Acinetobacter baumannii*, *Staphylococcus aureus*, and *Streptococcus pneumoniae* were obtained from patient samples at Chittaranjan National Cancer Institute (CNCI), Kolkata, India (provided by Dr. Subhranshu Mandal). Strain identification was performed using VITEK MALDI-TOF MS.

*E. coli*, *A. baumannii*, and *S. aureus* were cultured in Luria–Bertani (LB) broth at 37°C with shaking at 180 rpm under aerobic conditions. *S. pneumoniae*) was cultured in Tryptone Soy Broth (TSB) at 37°C under 5% CO_2_.

#### Cell lines

WRL68 (human hepatic cells) and HaCaT (human keratinocyte) cell lines were used. WRL68 cells were obtained from National Centre for Cell Science (NCCS), Pune, India, and HaCaT cells were obtained from ATCC (Cat# CRL-2404). HaCaT cells were authenticated by ATCC using short tandem repeat (STR) profiling, whereas WRL68 cells were not independently authenticated after acquisition, consistent with standard practice for this non-commercial cell line. All cells were verified for mycoplasma contamination. Cells were cultured in Dulbecco’s Modified Eagle Medium (DMEM) supplemented with 10% fetal bovine serum (FBS) and maintained under standard incubation conditions at 37°C in a humidified atmosphere containing 5% CO_2_. Cells were used within a limited number of passages after thawing to ensure reproducibility.

#### Experimental animals

BALB/c mice (male, 6–8 weeks old) were obtained from the Centre for Laboratory Animal Research and Training (CLART), Kalyani, India. A total of 15 mice were used. Mice were randomly assigned to three groups (n = 5 per group): control (saline treatment), guanosine hydrogel (G gel) and G4TZ hydrogel treatment. Animals were maintained under standard laboratory conditions (12 h light/dark cycle, 22 ± 2°C, 50–60% humidity).

All animal experiments were approved by the Institutional Animal Ethics Committee (IAEC) of the Indian Association for the Cultivation of Science (IACS/IAEC/s/2025/JD-03) and conducted in accordance with CPCSEA guidelines for the care and use of laboratory animals.

### Method details

#### Preparation of hydrogels

The guanosine gel was prepared by mixing guanosine, (10 mg, 0.035 mmol), phenylboronic acid, (4.3 mg, 0.035 mmol, 1.0 equiv.), and KOH (1.4 mg, 0.025 mmol, 0.7 equiv.) in 0.5 mL MilliQ water. The solution was heated to 80°C until a clear solution was obtained. The resulting solution was allowed to cool to room temperature and left undisturbed for gel formation.

The G4TZ-gel was prepared similarly by mixing guanosine, (10 mg, 0.035 mmol), phenylboronic acid (4.3 mg, 0.035 mmol, 1.0 equiv.), thiazole peptide (10μl, 10mM), KOH (1.4 mg, 0.025 mmol, 0.7 equiv.) in 0.5 mL MilliQ water. The solution was heated to 80°C until a clear solution was obtained. The resulting solution was allowed to cool to room temperature and left undisturbed for gel formation. A clear and strong gel was formed within 5 min. The gel was found to have pH ∼ 8.

#### Rheological study

Rheological experiments were performed using an Anton Paar MCR 102 Compact rheometer at 25°C. A parallel plate (PP 25) was used as the measuring system. The viscoelastic properties in terms of amplitude sweep tests were performed at a constant 10 rad/s angular frequency (ω) by sequentially applying low strain (0.05%) and high strain (100%) conditions at specific time gaps to ensure the complete gel-to-sol (G′ > G″) and sol-to-gel (G″ >G′) conversion.

#### Characterization of hydrogel

##### Circular dichroism analysis

Circular dichroism (CD) spectroscopic studies of the G4TZ hydrogel were performed in a JASCO J-815 spectropolarimeter using a quartz cell of 1 mm optical path length at 25°C. CD spectrum of the hydrogel was the average of three scans collected between 200 and 400 nm. The scanning speed of the CD instrument was 100 nm/min and the response time was 1s. The final analysis of the recorded spectra was conducted using OriginPro 8.0 (OriginLab Corp.).

##### Powder X-ray diffraction (PXRD) study

Powder X- ray Diffraction (PXRD) patterns of G4TZ, guanosine, phenylboronic acid and thiazole derivative was analyzed with a dried thin film of the gel on a glass slide using an X’Pert PRO X-ray Powder Diffractometer (PANalytical, Netherlands made) at 25°C.

##### Fluorescence spectroscopy

Fluorescence measurements were performed using a Horiba Fluorolog spectrofluorimeter at 25°C in thermostated cell holder. All measurements were carried out in a 1 mm path-length quartz microcuvette.

For ThT-binding assays, G4TZ hydrogel and G gel samples were added to 1 μM Thioflavin T solution prepared in 50 mM Tris–KCl buffer. The samples were excited at 450 nm, and emission spectra were recorded between 460–650 nm.

To evaluate the binding interaction between the thiazole peptide (TZ) and guanosine (G) gel fluorescence titration experiments were conducted. The concentration of TZ was fixed at 1 μM, while the concentration of G gel was varied in 50 mM Tris-KCl buffer. TZ was excited at its absorbance maximum (325 nm), and the emission spectra were recorded from 340 nm to 550 nm.

The binding constant was determined by fitting the fluorescence data using the Hill1 sigmoidal equation in Origin 2018 :

$$F=F_{0}+\frac{\left(F_{\max}-F_{0}\right)\left[G\right]}{K_{D}+\left[G\right]}$$

where *F* is the observed fluorescence intensity, *F*_*0*_ is the initial fluorescence, *F*_*max*_ is the maximum fluorescence intensity, *[G]* is the concentration of guanosine gel, and *K*_*D*_ is the apparent dissociation constant.

To examine the significance of the iminoboronate ester bond, an ARS (Alizarin Red S) binding assay was performed. Guanosine (G), phenylboronic acid derivative, and the bis-thiazole derivative were mixed with ARS in a stoichiometric ratio in the presence of aqueous KOH. ARS was excited at 470 nm, and emission spectra were collected over the range of 485–670 nm.

For drug release studies, an anticancer drug doxorubicin (Dox) was incorporated into the G4TZ hydrogel during gel formation. For the release study, 1 mL of buffer solution at pH 4.8, 7.4, and 8.5 was carefully added onto the Dox-loaded hydrogel and incubated at room temperature for 72 h. At defined time intervals, aliquots were collected to monitor drug release. The fluorescence intensity of Dox was measured at 490 nm to quantify the release over time.

##### FTIR study

For FTIR analysis, the G4TZ hydrogel (100 mM) was lyophilized, and spectra were recorded on a Shimadzu FTIR-8400S spectrometer in the 600–4000 cm^-1^ range using the KBr pellet technique. FTIR spectra of the individual components guanosine, boronic acid, and bis-thiazole amide were also recorded under identical conditions for comparison.

##### NMR study

For variable-temperature (VT) ^1^H and ^11^B NMR spectroscopy, the required amounts of guanosine, boronic acid, and bis-thiazole were dissolved in 500 μL of freshly prepared KOH (100 mM) in D_2_O within glass vials. The mixtures were heated to 80°C until clear solutions formed, then transferred into NMR tubes and cooled to room temperature to allow hydrogel formation. After gelation, VT ^1^H and ^11^B NMR spectra were recorded on a Bruker AVANCE 500 MHz spectrometer.

#### Morphological study

##### TEM and AFM analysis

For the TEM experiment, 5 mg of the G4TZ gel and G gel was diluted with 1 mL of MilliQ water to obtain a transparent dispersion. Then 10 μL of the sample was drop casted on the carbon coated copper grid followed by drying overnight in desiccator. The gel was analyzed using ultra high-resolution field emission gun transmission electron microscopy (UHR-FEGTEM), manufactured by JEOL 2100 KeV at 200 KeV.

The AFM was carried out on a NT-MDT in semi-contact mode. In order to obtain a transparent dispersion 1 mg of the G4TZ -gel was diluted with 1 mL of MilliQ water. 10 μL of aqueous hydrogel dispersion was drop casted onto the freshly cleaved mica (Agar Supplies) and allowed to dry in a desiccator for 16 h prior to imaging. The images were analyzed in Gatan and WSxM 5.0 software, respectively.

#### *In Cellulo* study

##### Antimicrobial activity

The antibacterial property of the hydrogels was assessed by determining minimum inhibitory concentration (MIC) using broth microdilution assay. Antibacterial properties of the hydrogels were determined against both Gram-positive (*S. aureus and S. pneumoniae)* and Gram-negative (*E. coli, Acinetobacter baumanii*) bacteria. Different bacterial strains were cultured for overnight at 37°C in suitable media containing ∼109 CFU mL^−1^. 1.5% of the overnight-grown primary culture was inoculated into fresh, appropriate media and incubated at 37°C with shaking at 180 rpm until reaching a turbidity of 0.5 MCF (log phase). The bacterial suspensions were then inoculated with increasing concentrations of the hydrogels in a 96 well microplate and incubated at 37°C for 24 h. After incubation, the optical density (OD) values were measured at 600 nm using a Spectra Max ID5 Multi-Mode Microplate Reader Platform. The experiment was performed in triplicates. The MIC value of the respective hydrogels was determined as the concentration at which no visible bacterial growth was observed.

##### Cell culture

WRL68 (human hepatic cells) cells and human keratinocyte (HaCaT) cells were cultured in DMEM containing high glucose (5.5 mM supplemented with 10 % FBS) at pH 7.4. Cells were maintained in tissue culture plates containing 4 × 10^5^ cells/well at 37°C in an atmosphere of 5 % carbon dioxide (CO_2_) for 24 h. To evaluate the *in vitro* effects of G4TZ, cells were treated with different concentrations of the hydrogel and incubated in the same condition for another 24 h for further investigation.

##### XTT assay

The XTT cell proliferation assay was employed to assess the cytotoxicity of G4TZ hydrogel. This assay is based on the ability of metabolically active cells to reduce the yellow tetrazolium salt, XTT [2,3-bis-(2-methoxy-4-nitro-5-sulfophenyl)-2H-tetrazolium-5-carboxanilide], into an orange-colored formazan dye. The reduction process is facilitated by the addition of PMS (N-methyl dibenzopyrazine methyl sulfate), which acts as an electron-coupling agent. This bioreduction is directly linked to the production of NAD(P)H during glycolysis and therefore occurs only in viable cells. For the assay, WRL 68 and HaCaT cells were seeded in 96-well plates and treated with varying concentrations of G4TZ for 24 hours. Following the incubation period, 25 μL of a freshly prepared solution containing 4 mL of XTT (1 mg/mL) and 10 μL of PMS (3 mg/mL) was added to each well containing 100 μL of cell culture medium. After further incubation, the absorbance of the resulting formazan dye was measured at 450 nm using a microplate reader. Cell viability was calculated using the following equation:

$$Viable\;cells\left(\%\right)=\frac{A\;of\;treated\;cells}{A\;of\;untreated\;cells}\times 100$$

where *A* represent the absorbance values of treated and untreated cells, respectively.

##### SEM analysis

*S. pneumoniae* was grown at mid log phase (0.5 McFarland standard) and incubated in the absence and presence of G4TZ gel at standard culture conditions for 24 hours. The bacterial cells were then centrifuged at 6000 rpm for 3 min and washed two times with PBS buffer. Next, the bacteria were fixed with 2.5% glutaraldehyde at 4°C for 1 h. They were then washed with PBS and dehydrated by washing in increasing concentrations of ethanol (50, 70, 80, 90, and 100% for 10 min) at room temperature. The samples were then drop casted on slides and mounted on gold coated aluminum stubs. The changes in morphology of the *S. pneumoniae* cells were visualized using a FESEM (JEOL, JSM 6700F) operating at 1 kV. Images were processed using ImageJ software.

##### Confocal imaging

The MDR *S. pneumoniae* was cultured overnight at 37°C and 180 rpm in fresh TSB. 1.5% of the overnight-grown primary culture was inoculated into fresh, appropriate media and incubated at 37°C with shaking at 180 rpm until reaching a turbidity of 0.5 MCF (log phase). Log phase cells were then treated with bis-thiazole amide and G4TZ hydrogel (less than MIC concentration) and kept overnight at 37°C. The cells were centrifuged at 6000 rpm for 5 min and washed with 1× PBS twice. Finally, the bacterial cell pellet was dissolved in 200 μl 1× PBS and mounted on glass slide. The sample was then visualized using Leica DMI8 fluorescence microscope.

##### Flow cytometry

Integrity of the bacteria membranes was quantified by Flow cytometry. *S. pneumoniae* was grown at mid log phase (0.5 McFarland standard) and incubated G4TZ hydrogel and kept overnight at 37°C. The cells were centrifuged at 6000 rpm for 5 min, washed with 1× PBS twice and diluted to 1× 10^6^ cells. Then 5 μg/mL PI and 3μM SYTO 9 was added and kept in dark for 15min. The data were recorded using FITC and PI filter in FACS Flow cytometer (BD Biosciences).

##### Live dead assay

Bacterial viability was assessed using a Live/Dead fluorescence assay using SYTO 9 and propidium iodide (PI) staining. Briefly, *S. pneumoniae* were grown to log phase and incubated with G4TZ hydrogel for 12hrs at 37°C. After treatment the cells were centrifuged at 6000 rpm for 5 min, washed with 1× PBS twice and stained with SYTO 9 and PI to final concentrations of 5 μM and 30 μM. Samples were incubated in the dark at room temperature for 15 min and imaged using fluorescence microscopy(Olympus IX73). SYTO 9 fluorescence was detected using 488 nm excitation with a 505–550 nm emission filter, while PI fluorescence was recorded using an excitation range of 540–580 nm and an emission range of 600–660 nm. Images were processed and analyzed using ImageJ software.

##### Bacterial viability assay

*S. pneumoniae* was grown at mid log phase (0.5 McFarland standard) and incubated with G gel, thiazole peptide (TZ), or G4TZ hydrogel. At 3, 6, 12, and 24 h post-treatment, bacterial suspensions were centrifuged at 6000 rpm for 5 min, washed twice with 1× PBS, and resuspended in PBS. 200 μL solutions was transferred to a 96 well plate, and bacterial viability was quantified using the SpectraMax ID5 Multi-Mode Microplate Reader by measuring absorbance at 600 nm (OD_600_).

##### Quantification of ROS accumulation

Intracellular ROS accumulation was measured using the DCFDA dye, a ROS indicator (Invitrogen, Burlington, Ontario, Canada). Bacterial cells treated with G gel, thiazole peptide (TZ), or G4TZ hydrogel were collected at 3, 6, 12, and 24 h post-treatment, centrifuged at 6000 rpm for 5 min, washed twice with 1× PBS, and resuspended in 500 μL PBS. Then the cells were incubated for 30 min in presence of DCFDA (1 μM). After washing with PBS, 200 μL of the Bacterial suspension was analyzed using Spectra Max ID5 Multi-Mode Microplate Reader Platform at excitation and emission wavelengths of 485 nm and 530 nm, respectively.

##### DNA fragmentation assay

*S. pneumoniae* was grown at mid log phase (0.5 McFarland standard) and incubated with varying concentrations of G4TZ gel and kept overnight at 37°C. The cells were centrifuged at 6000 rpm for 5 min and washed with 1× PBS twice. Then the bacterial pellet was resuspended in 1mL TE buffer and incubated for 30 mins at 37°C in the presence of 2 mg/mL RNase and 10 mg/mL lysozyme. Next, the cells were incubated with 10% SDS and 10 mg/mL proteinase K for 30 mins at 55°C. Then, DNA was extracted from the samples by phenol chloroform extraction and ethanol precipitation methods. Finally, 1% agarose gel electrophoresis in 0.5% TBE buffer was performed to assess the integrity of the bacterial genome.

##### Biofilm inhibition using AFM

*S. pneumoniae* was grown in tryptone soy broth (TSB) at 37°C for 24 h and then diluted into the TSB medium (10^5^ cells/mL). For each concentration of the hydrogel (1 and 5 μg/mL), 5 mL of bacterial suspension was incubated in a 6-well plate containing round glass cover slips for 24 h at 37°C. After adhesion, the media and planktonic cells were removed by rinsing the substrate two times with sterile PBS. Next, the bacteria were fixed with 2.5% glutaraldehyde at 4°C for 1 h. They were then washed with PBS air dried and observed using AFM. All AFM images were then processed to analyze the biofilm formation behavior using WSxM 4.0 software.

##### *In vitro* wound healing assay

HaCaT cells were grown to confluence in 6-well plates and a line-shaped scrape was made through the confluent monolayers using a plastic pipette tip. The cells were treated with G gel and G4TZ gel of (5μg/ml) and incubated in 37°C humidified incubator with 5% CO_2_ for 12 h. Several scratch areas were observed and photographed using Dewinter optical microscope at 6 h and 12 h after scratching and treatment.

##### Immunofluorescence

HaCaT cells were cultured on glass coverslips at 37°C and 5% CO_2_. Upon reaching the desired confluency, cells were treated with G4TZ and incubated overnight. Cells were then fixed with 4% paraformaldehyde for 15 min at room temperature, permeabilized with 0.1% Triton X-100 for 10 min, and blocked with 5% bovine serum albumin (BSA) in PBS for 1 h. Samples were incubated with a FITC-labeled anti-Ki67 antibody (1:100 dilution) overnight at 4°C. After washing with PBS, coverslips were mounted and imaged using a Leica DMi8 fluorescence microscope at 40× magnification.

##### *In vivo* wound healing assay

Full-thickness (epidermis, dermis, and subcutis) wounds were induced on the dorsal side of anesthetized BALB/c mice by using a sterile seizer (2cm length, 1cm width). Wounds were then treated with guanosine gel and G4TZ hydrogel, including a control group with saline. Changing hydrogel dressing was performed over 12 days. Wound healing progress was assessed by capturing photographs on days 3, 5, 8, and 12. The histopathological studies of skin was carried out from 10% formaldehyde fixed tissues of the healthy control, untreated and G4TZ treated groups, followed by haematoxylin-eosin(HE) staining of the paraffinized sections (5 μm). The slides were then imaged with Dewinter optical microscope to observe any histological abnormalities or changes in the treated groups.

### Quantification and statistical analysis

The data presentation is described in each figure legend according to the experimental design. Reactive oxygen species (ROS) levels and bacterial growth (OD_600_) were quantified at the indicated time points, and Quantitative data (Figures 4A and 4B) are presented as mean ± standard deviation (SD) from three independent experiments (*n* = 3), as indicated in the respective figure legends. Bar plots represent mean values, and error bars correspond to the standard deviation derived from biological replicates.

DNA fragmentation analysis (Figure 4C) was evaluated qualitatively based on agarose gel electrophoresis band integrity.

Atomic force microscopy (AFM) data (Figure 4D) were analyzed by extracting surface height and roughness parameters from topographical images. Height distribution histograms were used to assess biofilm structural heterogeneity. AFM data are presented as representative images with quantitative thickness and roughness values derived from *n* = 2 independent biofilm cultures analyzed using WSxM 4.0 software.

All data processing and graphing were performed using OriginPro 8.0, and image analyses were conducted using ImageJ where applicable.

## Supplemental information

Document S1. Figures S1–S11 and Scheme S1
