# Supplementary material for: G-quartet driven adaptive hydrogels that integrate constitutional dynamics for antibacterial action and tissue repair
Source: iScience. 2026 Jun 9;29(6):116107. doi: 10.1016/j.isci.2026.116107 (PMC13273585; doi:10.1016/j.isci.2026.116107)
Supplement: Document S1. Figures S1–S11 and Scheme S1 [file mmc1.pdf]

iScience, Volume *29*

Supplemental information

G-quartet driven adaptive hydrogels that integrate constitutional dynamics for antibacterial action and tissue repair

Sudeshna Mondal, Binayak Lala, Debasmita Biswas, Jean Marie Lehn, and Jyotirmayee Dash

Methods S1. Synthesis and characterization of thiazole peptide TZ. Related to Scheme 1 and STAR Methods.

1. General Synthesis Information:

All starting materials were obtained from commercial suppliers and used as received. All experiments were carried out under an inert atmosphere of argon in flame-dried apparatus, and the reaction mixture was magnetically stirred. Wherever needed solvents were dried using standard procedures. Products were purified by flash chromatography on silica gel (100–200 mesh, Merck). Unless otherwise stated, yields refer to analytical pure samples. NMR spectra were recorded in $CDCl_3$ unless otherwise stated. ^{1}H- NMR spectra were recorded at 500 MHz using Brüker AVANCE 500 MHz and JEOL 400 MHz instruments at 278 K. Signals are quoted as δ values in ppm. Data are reported as follows: chemical shift, integration, multiplicity (s = singlet, d = doublet, t = triplet, q = quartet, p = pentet, br = broad, m = multiplet) and coupling constants (Hz). ^{13}C-NMR spectra were recorded on either a JEOL-400 (100 MHz) or a Brüker AVANCE 500 MHz (125 MHz) with complete proton decoupling. Chemical shifts (δ) are reported in ppm downfield from tetramethylsilane with the solvent as the internal reference ($CDCl_3$: δ77.00 ppm). HRMS analyses were performed with Q-TOF YA263 high resolution (Water Corporation) instruments by +ve mode electrospray ionization. All general chemicals were purchased from Sigma-Aldrich.

2. Synthesis of thiazole peptide:

The thiazole peptide was synthesized via a stepwise amide coupling strategy. Thiazole amino acid building block **3** was first synthesized from thiourea **1** and bromoethyl acetoacetate **2**. Protection of compound **3** with Boc, followed by ester hydrolysis of the intermediate **4**, furnished the acid building block **5** (Scheme S1). Amide coupling between building blocks **3** and **5** was performed using HBTU and DIEA in anhydrous CH_2Cl_2, yielding the dipeptide **6**. Subsequent ester hydrolysis of Boc-dipeptide **6** generated acid **7**, which was further coupled with dodecylamine **8** to give the dodecyl-appended Boc-dimer thiazole peptide acid **9**. Final deprotection with TFA in CH_2Cl_2 afforded the target dimeric thiazole peptide **TZ** as its TFA salt.

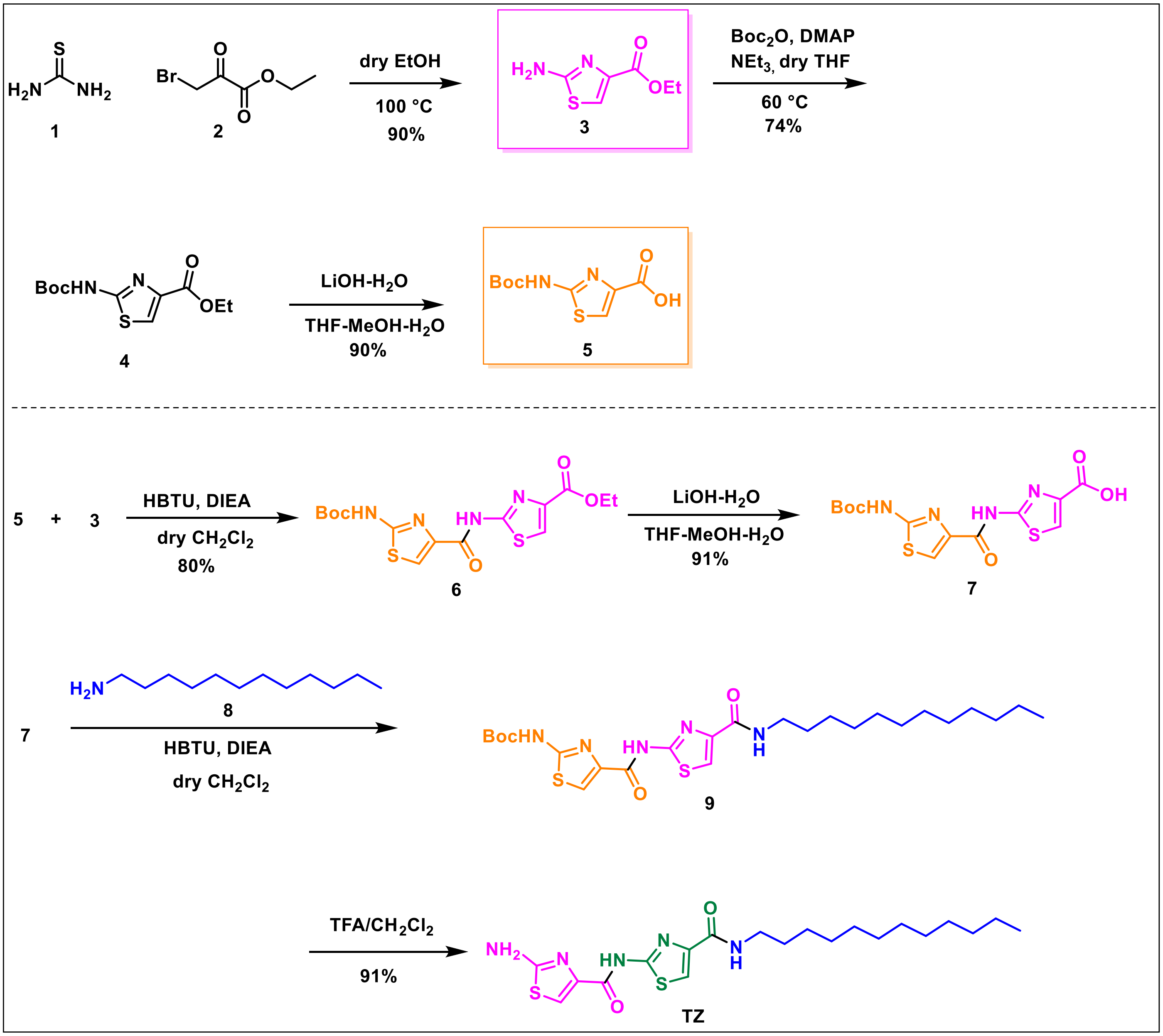

Scheme S1. Synthesis of thiazole peptide **TZ**. **Related to Scheme 1.**

Synthesis of thiazole amino acid building block 3 [S1]**:** Thiazole amino ester **3** was obtained in 90% yield by refluxing thiourea **1** and ethyl bromopyruvate **2** in dry ethanol at 100 °C. ^{1}H NMR (400 MHz, DMSO-*d6*): 7.45 (1H, s), 7.22 (2H, sbr), 4.19 (2H, q, J = 7.3 Hz), 1.25 (3H, t, J = 6.7 Hz); ^{13}C NMR (100 MHz, DMSO-*d6*): 168.2, 161.0, 142.2, 116.9, 60.1, 14.1; HRMS (ESI) calcd for $C_6H_9N_2O_2S$ [M+H]+: 173.0379; Found: 173.0391.

Synthesis of Boc-protected thiazole amino ester 4[S1]**:** To a stirred solution of compound **3** (2 g, 11.6 mmol) in dry THF (40 mL), triethylamine (2.12 mL, 15.2 mmol), DMAP (140 mg, 1.16 mmol), and di-tert-butyl dicarbonate (3 mL, 12.7 mmol) were added sequentially. The reaction mixture was heated at 60 °C for 1 hour, then cooled to room temperature (25 °C) and quenched with saturated aqueous ammonium chloride solution (50 mL). The resulting mixture was extracted

with ethyl acetate (3 × 20 mL). The combined organic extracts were dried over anhydrous sodium sulfate, filtered, and concentrated under reduced pressure. The crude product was purified by column chromatography to afford pure thiazolyl carbamate **4** as a white solid (2.34 g, 74%). ^{1}H NMR (400 MHz, DMSO-*d*$_6$): 11.77 (1H, s$_{br}$), 7.99 (1H, s), 4.25 (2H, q, *J* = 6.7 Hz), 1.47 (9H, s), 1.27 (3H, t, *J* = 7.3 Hz); ^{13}C NMR (100 MHz, DMSO-*d*$_6$): 160.9, 159.8, 153.0, 141.3, 122.2, 60.4, 27.8, 14.1; HRMS (ESI) calcd for $C_{11}H_{16}N_2O_4SNa$ [M+Na]$^+$: 295.0728; Found: 295.0728.

Synthesis of Boc-protected thiazole amino acid 5[S1]: To a stirred solution of Boc-protected thiazole ester **4** (2.0 g, 7.32 mmol) in THF/MeOH/H_2O (3:3:1, 30 mL), LiOH·H_2O (923 mg, 21.97 mmol) was added. The reaction mixture was stirred at room temperature for 3-4 h and monitored by TLC. Upon completion, the solvent was evaporated under reduced pressure. The resulting residue was dissolved in a small amount of water, and saturated $KHSO_4$ solution was added dropwise under cold conditions to precipitate the product. The white solid obtained was filtered and dried to yield thiazole amino acid **5** (1.6g, 90%). ^{1}H NMR (400 MHz, DMSO-*d*$_6$): 7.87 (1H, s), 1.45 (9H, s); ^{13}C NMR (100 MHz, DMSO-*d*$_6$): 162.7, 159.8, 153.3, 142.8, 121.9, 81.9, 28.1; HRMS (ESI) calcd for $C_9H_{12}N_2O_4SNa$ [M+Na]$^+$: 267.0415; Found: 267.0403.

Synthesis of Boc-protected thiazole dipeptide 6: To a stirred solution of thiazole amino acid **5** (1.0 g, 4.11 mmol) in dry CH_2Cl_2, HBTU (2.4 g, 6.17 mmol) was added, followed by the addition of N, N′-diisopropylethylamine (DIEA) (2.14 mL, 12.33 mmol) at 0 °C. After stirring for 10 minutes, a solution of thiazole amine **3** (707 mg, 4.11 mmol) in dry CH_2Cl_2 (20 mL) was added at the same temperature. The reaction mixture was stirred at room temperature for 16–24 h. Upon completion (monitored by TLC), the solvent was evaporated under reduced pressure. The residue was dissolved in ethyl acetate and sequentially washed with 1 N HCl, saturated $NaHCO_3$ solution, and brine. The organic layer was dried over anhydrous Na_2SO_4, filtered, and concentrated. The crude product was purified by column chromatography to afford dipeptide **6** as an off-white solid (1.3 g, 80%). ^{1}H NMR (500 MHz, $CDCl_3$): 10.68 (1H, s$_{br}$), 9.01 (1H, s$_{br}$), 7.85 (1H, s), 7.82 (1H, s), 4.27 (2H, q, *J* = 7.3 Hz), 1.47 (9H, s), 1.33 (3H, t, *J* = 6.7 Hz); ^{13}C NMR (125 MHz, $CDCl_3$): 161.5, 160.2, 159.2, 157.8, 152.3, 142.5, 142.0, 122.5, 120.5, 83.3, 61.4, 28.2, 14.3; HRMS (ESI) calcd for $C_{15}H_{19}N_4O_5S_2$ [M+H]$^+$: 399.0791; Found: 399.0778.

Synthesis of Boc-protected thiazole dimeric acid 7: To a stirred solution of Boc-protected dipeptide **6** (1.0 g, 2.52 mmol) in THF/MeOH/H_2O (3:3:1, 20 mL), LiOH·H_2O (317 mg, 7.53 mmol) was added at 0 °C. The reaction mixture was then stirred at room temperature for 3-4 hours and monitored by TLC. Upon completion, the solvent was evaporated under reduced pressure. The crude residue was dissolved in a small amount of water, and saturated $KHSO_4$ solution was added dropwise under cold conditions. This resulted in the precipitation of thiazole dimeric acid **7** as a white solid (0.8 g, 91%). ^{1}H NMR (500 MHz, DMSO-*d*$_6$): 12.29 (1H, s$_{br}$), 11.82 (1H, s$_{br}$), 8.20 (1H, s), 8.03 (1H, s), 1.49 (9H, s); ^{13}C NMR (100 MHz, DMSO-*d*$_6$): 162.4, 160.1, 159.4, 157.7, 142.4, 142.3, 122.9, 120.5, 81.9, 27.9; HRMS (ESI) calcd for $C_{13}H_{14}N_4O_5S_2Na$ [M+Na]$^+$: 393.0303; Found: 393.0289.

Synthesis of thiazole peptide TZ: A solution of Boc-protected thiazole dimeric acid **7** (200 mg, 0.54 mmol) in dry DCM (20 mL) was treated with HBTU (1.54 g, 0.81 mmol), DIEA (0.3 mL, 1.62 mmol), and dodecylamine **8** (0.15 mL, 0.64 mmol). The reaction mixture was stirred at room temperature for 16–24 h. Upon completion (monitored by TLC), the

solvent was removed under reduced pressure. The crude residue was dissolved in ethyl acetate, and the organic layer was washed successively with 1 N HCl, saturated $NaHCO_3$ solution, and brine. The organic phase was dried over Na_2SO_4, filtered, and concentrated. Subsequently, the crude product **9** was directly used for the synthesis of **TZ**.

Boc-protected crude thiazole peptide **9** (130 mg, 0.242 mmol) was dissolved in CH_2Cl_2 (1 mL) and cooled to 0 °C. Trifluoroacetic acid (TFA) (1 mL) was then added, and the solution was allowed to warm to room temperature. The reaction mixture was stirred for 3–4 h and monitored by TLC. Upon completion, the solvent was removed under vacuum, and the residue was washed with cold diethyl ether twice which resulted in the formation of product **TZ**. The solid product was dried under reduced pressure to afford dodecyl-appended thiazole dipeptide **TZ** as a white solid (95 mg, 91%). ^{1}H NMR (500 MHz, DMSO-d_6) δ 11.29 (s, 1H), 8.12 (t, J = 6.0 Hz, 1H), 7.82 (s, 1H), 7.72 (s, 1H), 4.43 (s, 6H), 1.50 (t, J = 7.0 Hz, 2H), 1.23 (s, 16H), 0.84 (t, J = 6.7 Hz, 3H); ^{13}C NMR (125 MHz, DMSO-d_6) δ 169.2, 160.9, 158.8, 157.4, 145.4, 141.8, 118.1, 115.8, 31.8, 29.6, 29.5, 29.5, 29.5, 29.2, 29.2, 26.9, 22.6, 14.4. HRMS (ESI) calculated for $C_{20}H_{31}N_5O_2S_2$ $[M+H]^+$: 437.1919; Found 438.1998.

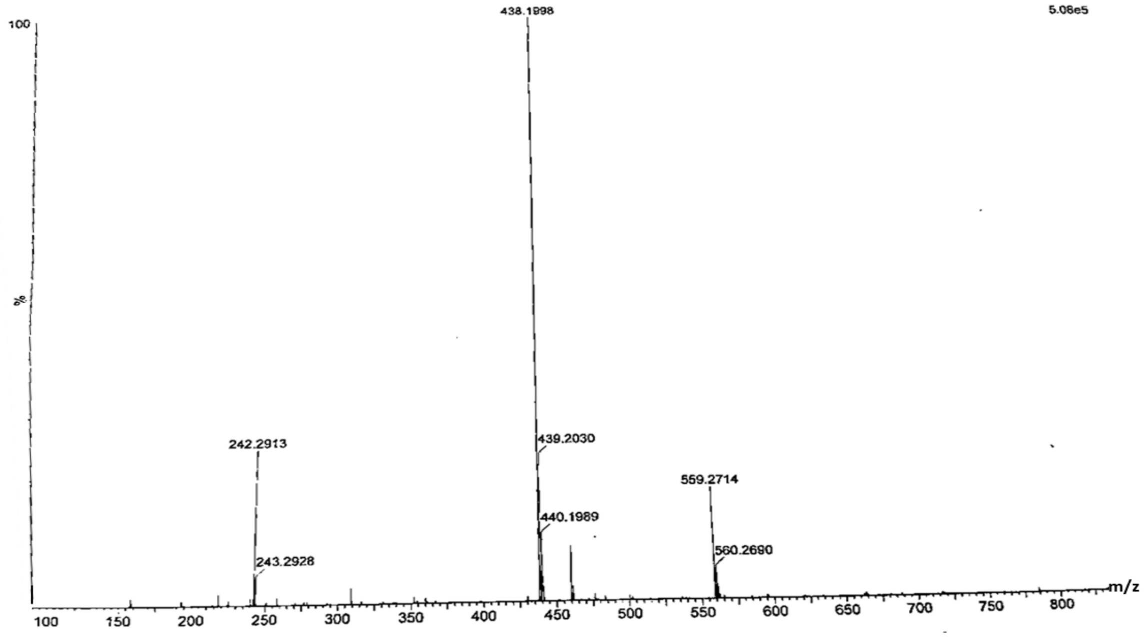

Figure S1. ESI-MS analysis of TZ.

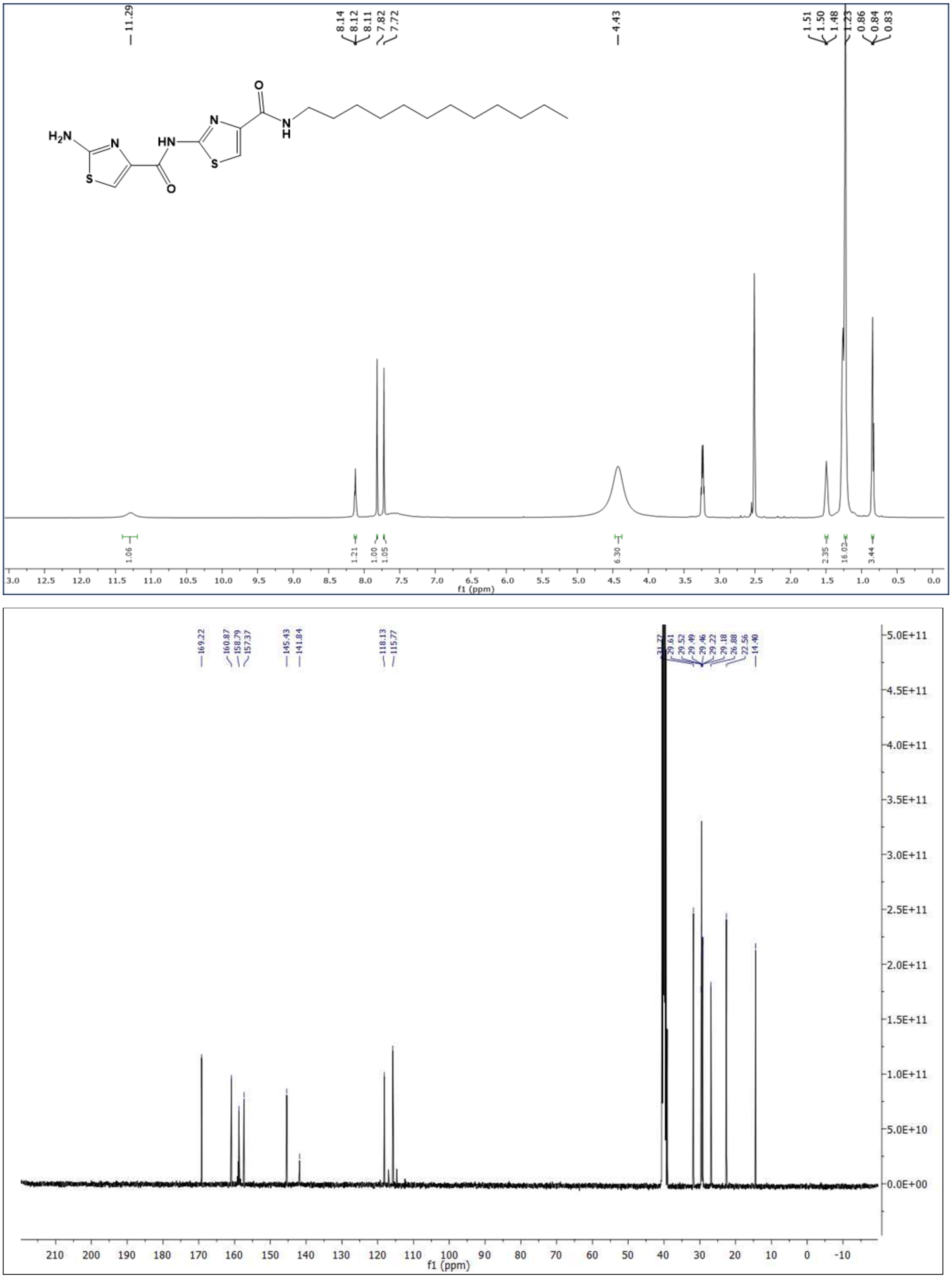

Figure S2. ^{1}H and ^{13}C NMR of thiazole peptide (TZ).

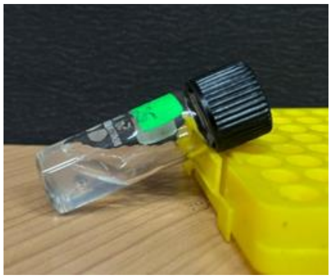

Figure S3. Related to STAR Methods. The hydrogel formed from guanosine, phenylboronic acid, and KOH was relatively less stable and gradually flowed downward upon inverting the vial.

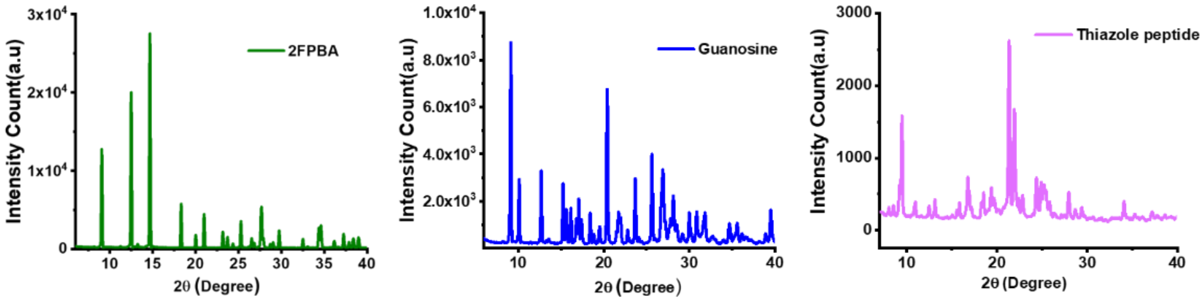

Figure S4. Related to Figure 1E. PXRD pattern of phenyl boronic acid derivative, guanosine and bis-thiazole (TZ).

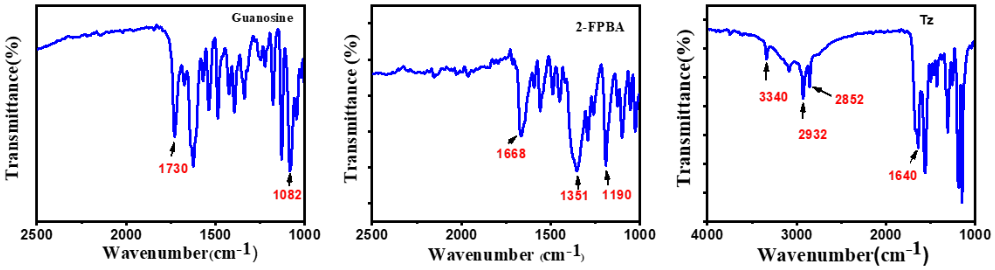

Figure S5. Related to Figure 2B, STAR Methods. FTIR spectra of phenyl boronic acid derivative, guanosine and bis-thiazole (TZ).

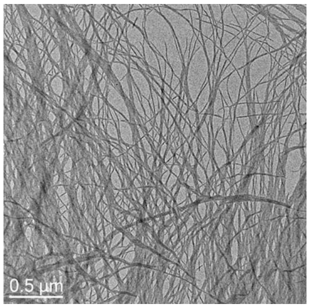

Figure S6. Related to Figure 2. TEM image of guanosine gel.

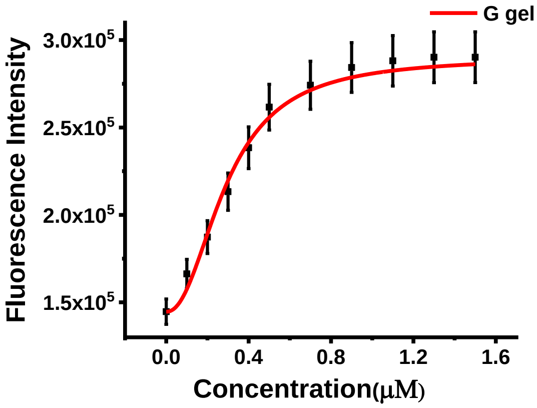

Figure S7. Related to Figure 2G. Hill1 sigmoidal plot indicating a high binding affinity of TZ for guanosine gel.

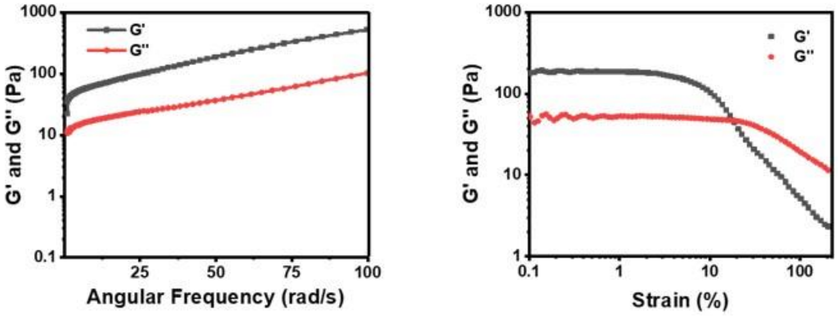

Figure S8. Related to Figure 1. Rheology for G gel, left panel frequency sweep, right panel amplitude sweep.

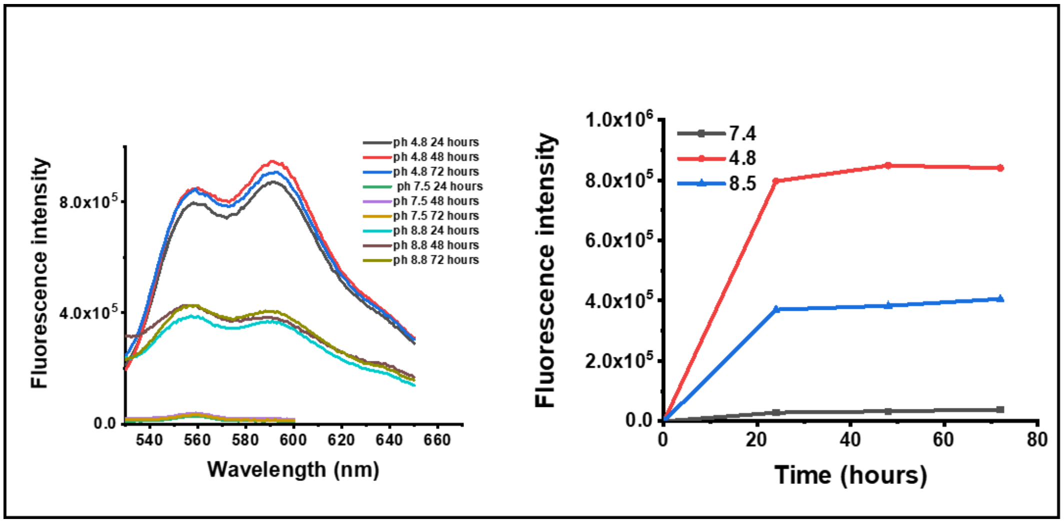

Figure S9. Related to STAR Methods. Left panel Fluorescence spectra of Dox loaded G4TZ hydrogel at pH 4.8, 7.4, and 8.5. Right panel Release profile of DOX from the G4TZ hydrogel at different time point and pH 4.8, 7.4, and 8.5.

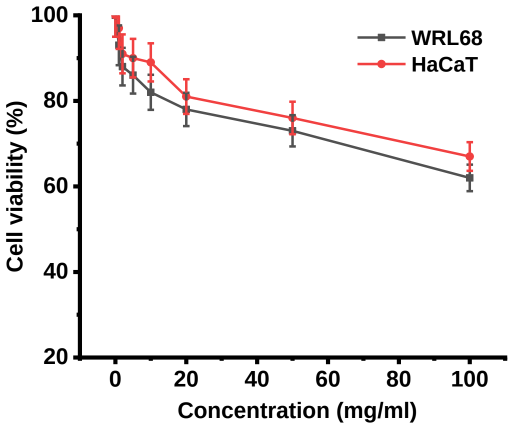

Figure S10. Related to STAR Methods. Cytotoxicity assay of G4TZ hydrogel in WRL 68 and HaCaT cell lines.

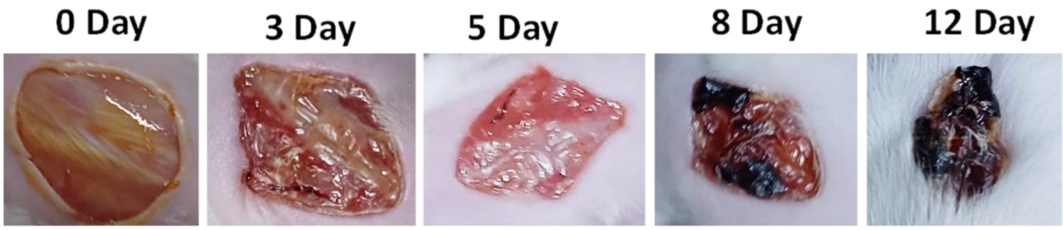

Figure S11. Related to Figure 5. *In vivo* wound healing evaluation. Images of wounds of guanosine gel (G gel) treated groups on days 0, 3, 5, 8 and 12.

Reference

S1. Dutta, D., Debnath, M., Müller, D., Paul, R., Das, T., Bessi, I., Schwalbe, H., and Dash, J. (2018). Cell penetrating thiazole peptides inhibit c-MYC expression via site-specific targeting of c-MYC G-quadruplex. Nucleic acids research *46*, 5355-5365.
